# Designing a Multi-Epitope Vaccine Candidate Against *Rhodococcus equi* Based on the Bioinformatics Technique

**DOI:** 10.3390/vetsci13070655

**Published:** 2026-07-07

**Authors:** Shiwen Gao, Guoqing Li, Xiangyu Wang, Weifang Gu, Dingnuoya Guo, Zongping Xian, Xuelian Ma, Jun Meng, Hongqiong Zhao, Lu Liu

**Affiliations:** 1 Xinjiang Key Laboratory of New Drug Research and Development for Herbivores, College of Veterinary Medicine, Xinjiang Agricultural University, Urumqi 830052, China; 16699188629@163.com (S.G.); 18116851185@163.com (G.L.); wang20010404yu@163.com (X.W.); weifang_gu@sina.com (W.G.); guodingnuoya@outlook.com (D.G.); m17799136761@163.com (Z.X.); maxuelian@xjau.edu.cn (X.M.); zhaohongqiong@sina.com (H.Z.); 2 College of Animal Science, Xinjiang Agricultural University, Urumqi 830052, China; junm86@xjau.edu.cn

**Keywords:** *Rhodococcus equi*, bioinformatics, antigen, multiple-epitope vaccine, immunogenicity

## Abstract

*Rhodococcus equi* (*R. equi*) primarily induces fatal pulmonary and extrapulmonary pyogenic granulomatous infections in foals, imposing substantial economic burdens on the equine industry. The emergence and spread of multidrug-resistant (MDR) *R. equi* have led to a therapeutic impasse in clinical settings. Although vaccination is a proven strategy against MDR pathogens, no commercial vaccine is currently available for *R. equi*. In this study, we employed a bioinformatics approach to systematically identify and prioritize antigenic epitopes derived from *R. equi* for multi-epitope vaccine design. Using ABCPred, NetMHCpan EL, and IEDB servers, 27 MHC-I and 9 MHC-II epitopes were selected from five previously validated *R. equi* vaccine candidates: ABC transporter, PBD2, NlpC/P60, Esterase, and M23. These epitopes were coupled with distinct peptide linkers to construct six multi-epitope vaccine constructs, designated V1–V6. The physicochemical properties, antigenicity, immunogenicity, and toxicity of the six vaccine constructs were analyzed, and the V3 and V4 constructs were ultimately selected. Using the HDOCK and Gromacs tools, the intermolecular interactions, binding affinity, and thermal stability of the V3 and V4 constructs with the equine MHC molecules EQCA-I and EQCA-II were evaluated. The results confirm that V3 and V4 exhibit strong binding affinity to EQCA-I and EQCA-II, with stable conformations following binding, indicating theoretical potential to induce humoral and cellular immunity in foals. Recombinant plasmids for V3 and V4 were constructed, and the V3 and V4 proteins were successfully prepared, confirming the feasibility of prokaryotic expression for these vaccine constructs. Immunization assays in SPF BALB/c mice showed that the multi-epitope vaccines elicited robust antigen-specific IgG antibody responses, reflecting preliminary humoral immunogenicity. However, these murine data have translational limitations, as they cannot fully represent equine immune responses. The findings establish a crucial theoretical foundation for the advancement of vaccines targeting *R. equi* while offering a reference for the design of vaccines against other drug-resistant microbial pathogens.

## Simple Summary

*Rhodococcus equi* is a type of bacteria that causes fatal lung and body-wide infections in foals (baby horses), creating severe economic burdens for the global equine breeding industry. As this bacteria species has grown increasingly resistant to common antibiotics, there are no reliable treatments or any approved commercial vaccines to prevent the infections it causes. Our study aimed to develop safe and effective vaccine candidates against this pathogen. We employed computer-aided design to screen protective vaccine components, identified two optimal candidates, and successfully prepared stable, highly producible vaccine proteins. Laboratory experiments verified that these candidates elicit robust antigen-specific IgG responses in mice. It should be noted that the current data only reflect preliminary humoral immunogenicity; the actual protective efficacy and cellular immune responses of the vaccines require further validation in subsequent studies. Our findings lay a critical foundation for developing *Rhodococcus equi* vaccines and provide a useful reference for designing vaccines against other drug-resistant bacteria.

## 1. Introduction

*Rhodococcus equi* (*R. equi*) is an opportunistic zoonotic pathogen known to primarily cause severe pyogranulomatous infections in both foals and immunocompromised individuals [1]. In horse stables or farms experiencing *R. equi* outbreaks, the incidence rate typically ranges from 20% to 40%, while the mortality rate after disease onset can reach 70–80% [2,3]. Apart from horses, *R. equi* also infects pigs, cattle, and sheep and poses a significant risk to immunocompromised humans, particularly those with HIV, where the infection rate is about 54.5% [4]. The bacterium’s ability to persist and multiply within macrophages facilitates the development of latent infections [5]. *R. equi* was acknowledged by the European Food Safety Authority (EFSA) as a key bacterial species of concern regarding antimicrobials in horses within the EU in 2022 [6].

Vaccines are the mainstay of defense and control against multidrug-resistant pathogens; however, to date, there is no approved commercial vaccine against *R. equi*. In 2009, the US approved horse immune plasma (HIP) for *R. equi* pneumonia prophylaxis on endemic farms, but the optimal prophylaxis age and minimum effective HIP infusion dose for foals remain undefined [7]. The protective efficacy of live-attenuated *R. equi* vaccines is well documented, yet replication-competent vaccine strains pose notable biosafety risks for clinical translation [8,9]. Electron beam (eBeam)-inactivated *R. equi* vaccines were developed to address safety concerns, with initial studies reporting preserved cell wall integrity and immunogenic potential [10]. However, subsequent trials showed that eBeam-inactivated vaccines failed to elicit sufficient immunogenicity or protect foals against virulent *R. equi* Challenge [11]. Only a limited number of *R. equi* antigens have been reported and experimentally validated to date, with virulence-associated proteins (Vaps) being the most well-characterized candidates for anti-*R. equi* vaccine development [12,13,14]. Given the intact cellular structure of *R. equi*, single-antigen vaccines generally underperform compared to inactivated or live-attenuated vaccines. Consequently, screening additional protective antigens to develop a multi-epitope vaccine targeting multiple *R. equi* epitopes represents a more effective strategy.

Multi-epitope vaccines, an emerging alternative to traditional vaccines, specifically elicit CD4^+^ and CD8^+^ T-cell responses targeting pre-selected protective epitopes, eliminating adverse reactions induced by irrelevant non-protective epitopes in intact antigens [15]. Unlike conventional vaccines carrying irrelevant pathogenic components, multi-epitope vaccines only retain core immunogenic epitopes, avoiding associated adverse reactions [16]. Multi-epitope vaccines can be rapidly produced via recombinant DNA technology or solid-phase peptide synthesis, eliminating the need for pathogen culture or native antigen purification and greatly shortening development timelines. Heng et al. [17] computationally engineered an influenza multi-epitope vaccine using immunoinformatic platforms, demonstrating potent induction of both humoral and cellular immunity. In a similar study, Vikas Kaushik et al. [18]. produced a tetravalent dengue vaccine construct that elicited cross-serotype antibody responses in rabbits, confirming its broad immunogenic potential. Similarly, Shan Ren et al. [19] created a multi-epitope vaccine targeting Acinetobacter baumannii, which induced high antibody titers and provided significant protection against bacteria challenges in murine models.

In previous studies, we screened out 12 *R. equi* vaccine candidates and validated the immunogenicity of five of them—ABC transporter substrate-binding protein (ABC transporter), penicillin-binding protein 2 (PBD2), NlpC/P60 family protein (NlpC/P60), esterase family protein (esterase), and M23 family metallopeptidase (M23)—through mouse immunization trials [20,21]. In this study, multiple bioinformatics tools were employed to identify dominant B- and T-cell epitopes and to construct an antigenic epitope library. This library was then used to assemble a multi-epitope vaccine based on the five selected *R. equi* vaccine candidates.

Candidate multi-epitope vaccines were first screened via antigenicity, immunogenicity and stability prediction, and their binding affinity to equine EQCA molecules was further evaluated via molecular docking and MD simulation. After codon optimization, we further constructed V3-pET-30a(+) and V4-pET-30a(+) recombinant plasmids, which were prokaryotically expressed by *E. coli* BL21(DE3). The expressed and purified target proteins were emulsified with adjuvants for mouse immunization to evaluate their ability to elicit antigen-specific antibody responses, providing theoretical data for research on *R. equi* multi-epitope vaccines.

## 2. Materials and Methods

We engineered a multi-epitope vaccine against *R. equi* infection utilizing bioinformatics. The schematic workflow shown in Figure S1 delineates the methodology integrated for vaccine architecture assembly.

### 2.1. Antigen Selection

In our previous studies, we validated 5 *R.equi* vaccine candidates—ABC transporter, PBD2, NlpC/P60, Esterase, and M23—through mouse immunization trials, all of which demonstrated good immunogenicity [20,21].

### 2.2. Prediction of Antigenic Epitopes

B-cell and T-cell epitopes play a critical role in immune responses. We employed multiple bioinformatics tools to predict epitopes for multi-epitope vaccine design. ABCPred https://webs.iiitd.edu.in/raghava/abcpred/index.html (accessed on 10 February 2024) was utilized to predict linear B-cell epitopes in potential vaccine candidates. This server utilizes sophisticated artificial neural networks, and predictions were made with a threshold value above 0.8 [22]. The 20-mer B-cell epitopes of candidate antigens were analyzed using IEDB’s NetMHCpan EL v.2.22, http://tools.iedb.org/mhci/ (accessed on 15 February 2024) and MHC-II v.2.22, http://tools.iedb.org/mhcii/ (accessed on 15 February 2024) tools [23,24]. T-cell epitopes binding to MHC-I and II alleles were subsequently analyzed using B-cell epitope data. Antigenic epitopes with MHC-I pre-rank ≤ 0.2 and MHC-II pre-rank ≤ 10 were selected.

T-cell epitopes require CD4^+^ or CD8^+^ T lymphocyte activation to elicit immune responses. The immunogenicity of screened T-cell epitopes was evaluated using IEDB tools http://tools.iedb.org/immunogenicity/ (accessed on 18 February 2024) http://tools.iedb.org/CD4episcore/ (accessed on 18 February 2024). We selected T-cell epitopes with MHC I immunogenicity scores above 0 and CD4^+^ T-cell composite scores below 60.

### 2.3. Assembly of the Multi-Epitope Vaccine Constructs

The final sequence of the multi-epitope vaccine was crafted by combining the epitopes identified through bioinformatics tools alongside suitable adjuvants and linker segments. In the present study, six multi-epitope vaccine candidates targeting *R. equi* were created and named V1-V6, respectively. The assembly processes for V1, V2, and V3 involve merging the MHC-I epitopes derived from all of the proteins, followed by the unification of MHC-II epitopes from the complete set of proteins. The assembly methodology for V4, V5, and V6 involves linking the MHC-I and MHC-II epitopes of each protein, followed by the sequential connection of the combined epitopes from the five proteins. Each vaccine formulation included the protein adjuvants beta defensin adjuvant, heparin-bound hemagglutinin adhesion (HBHA), and L7/L12 ribosomal protein [25], with the sequence of adjuvants, MHC-I epitopes, and MHC-II epitopes linked together utilizing specific linkers (a GGGS linker for the MHC-I epitope, a GPGPG linker for the MHC-II epitope, and an EAAAK linker for the adjuvant sequence). To boost antibody responses, a generic adjuvant T-cell epitope, Pan HLADR-binding epitope (PADRE), was also added, as it helps induce CD4^+^ T-cells, which enhances peptide vaccine efficacy [26]. A HEYGAEALERAG linker distinguished MHC-I and MHC-II epitope segments, and the 6 His tag sequence was added to the C-terminus to enable the purification and expression of the protein.

### 2.4. Assessment of Antigenicity, Solubility, Toxins, Allergenicity, and Physicochemical Properties of Multi-Epitope Vaccine Constructs

VaxiJen v.2.0 https://www.ddg-pharmfac.net/vaxijen/VaxiJen/VaxiJen.html (accessed on 23 February 2024) was used to predict antigenicity with a threshold of >0.4 [27]; AlgPred v.2.0 https://webs.iiitd.edu.in/raghava/algpred2/batch.html (accessed on 25 February 2024) was used to assess allergenicity, utilizing a cutoff value of −0.4 and achieving an accuracy of 85% [27]. The Protein-sol tool https://protein-sol.manchester.ac.uk/ (accessed on 25 February 2024) was used to predict solubility, with scores greater than 0.45 indicating good solubility [28]. ToxinPred2 https://webs.iiitd.edu.in/raghava/toxinpred2/batch.html (accessed on 26 February 2024) was used to evaluate toxicity [29], and AllerTOP v.2.0 https://www.ddg-pharmfac.net/allertop_test/ (accessed on 26 February 2024) was used to predict allergenicity [30].

Expasy ProtParam https://web.expasy.org/protparam (accessed on 27 February 2024) was used to predict physicochemical characteristics. This assessment involved determining the theoretical isoelectric point (pI), instability index, aliphatic index, half-life, and grand average of hydropathicity (GRAVY) of the vaccine constructs. Proteins with instability indices below 35 were deemed to exhibit antigenic stability [31].

### 2.5. Structure Prediction and Refinement of Multi-Epitopes Vaccine Constructs

The secondary structure was predicted using the PSIPRED tool http://bioinf.cs.ucl.ac.uk/psipred (accessed on 2 March 2024) [32], the 3D structures were predicted using the I-TASSER server https://zhanggroup.org/I-TASSER/ (accessed on 7 March 2024), and the optimal prediction model was selected based on its confidence score (C-score, −5, 2) [33]. GalaxyRefine https://galaxy.seoklab.org/cgi-bin/submit.cgi?type=REFINE (accessed on 10 March 2024) was used to polish the optimal prediction mode [34]. The refined 3D structures were validated on the SAVE and ProSA web servers. The SAVE server https://saves.mbi.ucla.edu/ (accessed on 13 March 2024) evaluates the overall protein quality by generating a Ramachandran plot [35], and the ProSA server https://prosa.services.came.sbg.ac.at/prosa.php/ (accessed on 15 March 2024) provides a Z-score to assess the quality of the tertiary structural model of the vaccine constructs, where a positive Z-score suggests the presence of questionable and unstable regions in the generated tertiary structural model [36].

### 2.6. Prediction of B-Cell Conformational Epitopes

In multi-epitope vaccine design, accurately predicting B-cell conformational epitopes is a critical factor for inducing strong antibody responses, ultimately determining the immunogenicity and protective efficacy of the vaccine. The ElliPro server http://tools.iedb.org/ellipro/ (accessed on 16 March 2024) is used to predict refined three-dimensional structures of B-cell conformational epitopes [37]. ElliPro assigns a score to each predicted epitope and selects B-cell conformational epitopes with scores greater than 0.6.

### 2.7. Molecular Docking of Multi-Epitope Vaccine Constructs with EQCA

Vaccine constructs and EQCA I/II protein structures were computationally refined and structurally optimized using Molecular Operating Environment 2019.1 (MOE 2019.1) software. The Amber10 force field was chosen, and the process included H_2_O and ion removal, protonation, insertion of lost atoms, finishing of incomplete sets, and protein energy minimization [38]. The HDOCK server http://hdock.phys.hust.edu.cn/ (accessed on 25 March 2024) was used to evaluate intermolecular interactions, binding affinity, and thermal stability between multi-epitope vaccine constructs and EQCA-I (PDB ID: Q0R0C1, Q0R0D4) and EQCA-II (PBD ID: KM9C44, KM9C51). Structural outputs underwent PyMOL v2.1 visualization for model analysis.

### 2.8. Molecular Dynamics Simulation and Analysis

To elucidate the binding mechanism of multi-epitope vaccine constructs to EQCA molecules, molecular dynamics (MD) simulations were performed on screened receptor protein–small molecule complexes using Gromacs 2020. Proteins were parameterized with the AMBER99SB-ILDN force field, and the TIP3P water model was employed, ensuring a minimum distance of 1.0 nm between protein atoms and the water box edge. System charge neutralization was achieved by adding sodium or chloride ions based on docking results. The MD protocol comprised four stages: energy minimization, heating, equilibration, and production simulation. Initial minimization (5000 steepest descent followed by 5000 conjugate gradient) constrained protein (and small molecule) heavy atoms while relaxing water. Subsequently, the entire system underwent minimization without constraints (5000 steepest descent; 5000 conjugate gradient). Following minimization, the system was gradually heated to 300 K over 50 ps and then equilibrated for 50 ps under NPT conditions. Finally, a 100 ns production MD simulation was conducted in the NPT ensemble. Trajectories were saved every 10 ps. Correlation analysis was conducted using the trjconv module, and ligand–protein binding free energies were calculated using the gmx_MMPBSA method within Gromacs 2020 [39]. Protein dynamics were characterized by Root Mean Square Deviation (RMSD), Root Mean Square Fluctuation (RMSF), Radius of Gyration (Rg), and Solvent-Accessible Surface Area (SASA). To elucidate protein–protein binding mechanisms, hydrogen bond formation was quantified throughout the simulation trajectory.

### 2.9. Prokaryotic Expression and Purification of Vaccine Constructs

The vaccine construct amino acid sequence was reverse-translated and optimized using GenScript’s online codon optimization tool https://www.genscript.com.cn/tools/gensmart-codon-optimization (accessed on 2 April 2024). *Hind* III and *Nde* I restriction enzyme sites were inserted into the optimized DNA sequence to construct a recombinant plasmid, which was then introduced into *E. coli* BL21(DE3). After incubation at 37 °C with shaking, Kana^+^ (30 μg/mL)—resistant liquid LB medium was inoculated and incubated until the OD_600nm_ value was about 0.6, and then IPTG was added to 1 mmol/L to induce expression for 6 h. After induction expression, cell disruption was performed using a low-temperature high-pressure continuous flow cell disruptor (JN-6.0C, Guangzhou Juneng Nano Biotechnology Co., Ltd., Guangzhou, China) at 4 °C and 1440 bar for 5–8 min until the solution became clear and non-viscous; protein purification was performed using a His-tag Purification Resin (degeneration-resistant) purification kit (P2229S, Beyotime, Shanghai, China).

### 2.10. Experiment on Immunization of BALB/c Mice with Multi-Epitope Vaccine

To evaluate immunogenicity, purified multi-epitope vaccines V3 and V4 were adjusted to 1.0 mg/mL for prime immunization and 0.5 mg/mL for booster immunization, respectively. Antigens were emulsified at 1:1 (*v*/*v*) with Freund’s Complete Adjuvant (FCA, for prime) or Freund’s Incomplete Adjuvant (FIA, for booster). Water-in-oil (W/O) emulsions were prepared under a low temperature following a combined vortex–tissue homogenizer procedure.

A total of 52 female BALB/c mice were randomly allocated to three groups (n = 18/group): the V3 immunization group, the V4 immunization group, and the adjuvant PBS control group. Immunization was administered via multi-point subcutaneous (s.c.) injection on the dorsum: prime immunization used 200 μL of emulsion containing 100 μg of the corresponding antigen mixed with FCA; two booster immunizations were given at 14-day intervals, each using a 200 μL emulsion containing 50 μg of the corresponding antigen mixed with FIA. The control group received 200 μL of adjuvant-mixed PBS per injection throughout the regimen. Serum samples were collected every 7 days from day 0 to 42 via tail bleeding to measure antibody titers.

## 3. Results

### 3.1. Antigenic Epitope Prediction Results 

A total of 64 B-cell, 623 MHC-I (9-mer), and 518 MHC-II (15-mer) epitopes were identified (Table S1). The screened MHC-I and MHC-II epitopes exhibited duplicate fragments at both the start and end sites. We opted to consolidate these duplicates into a single epitope. Twenty-seven MHC-I and nine MHC-II epitopes were identified as candidate epitopes for the subsequent construction of a vaccine (Table 1).

### 3.2. Assembly Results of Multi-Epitope Vaccine Constructs

**Figure 1:**
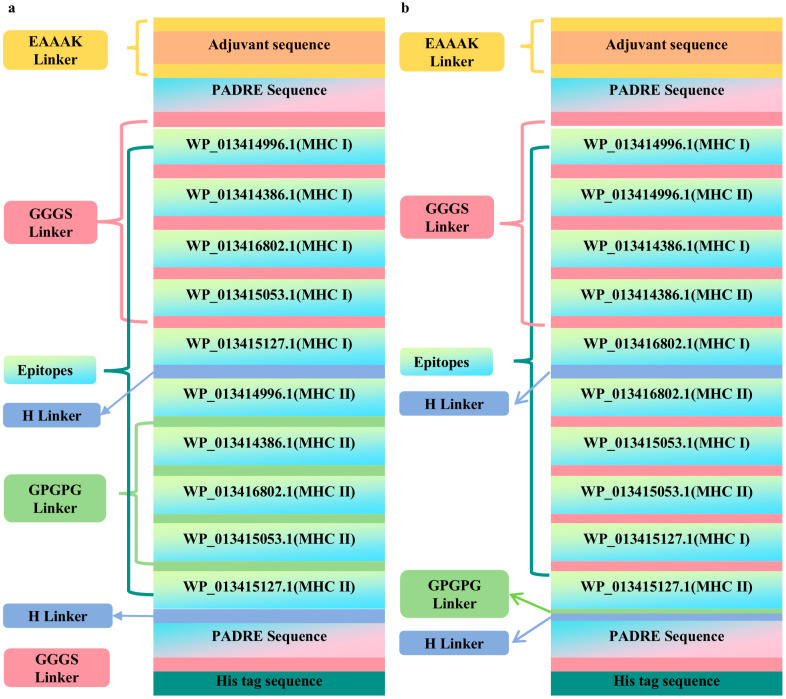
Diagram of two designs for multi-epitope vaccine constructs. (**a**,**b**) Yellow indicates EAAAK linker, pink indicates GGGS linker, green indicates GPGPG linker, blue indicates H linker, and teal indicates epitope.

Twenty-seven MHC-I epitopes and nine MHC-II epitopes were selected for the construction of a multi-epitope vaccine. Six multi-epitope vaccine constructs were designed and labeled V1–V6. The number of amino acid residues ranged from 600 to 800, and the molecular weight ranged from 60 to 80 kDa (Table S2), with each residue comprising protein adjuvants (including β-defensin proteins, HABA proteins, and L7/L12 ribosomal proteins), PADRE sequences, MHC-I epitopes, MHC-II epitopes, and linkers. The selected epitopes were separated using linkers (GGGS and GPGPG), and the initial adjuvant sequence was ligated to the PADRE sequence using the EAAAK sequence (Figure 1).

### 3.3. Evaluation of Antigenicity, Immunogenicity, Solubility, Toxicity, Allergenicity and Physicochemical Properties of Vaccine Constructs

The in silico evaluation of antigenicity, immunogenicity, solubility, toxicity, allergenicity, and physicochemical properties revealed that V2 exhibited a solubility score below the predefined threshold of 0.4, indicating poor recombinant expression potential. Consequently, V2 was excluded from further experimental validation. The remaining five multi-epitope fusion proteins (V1 and V3–V6) satisfied key vaccine design criteria, including a VaxiJen antigenicity score > 0.4, an instability index < 40 (indicating structural stability), and a negative grand average of hydropathicity (GRAVY) score (indicating hydrophilicity). These constructs were therefore advanced to subsequent molecular docking and dynamics simulation evaluations (Table 2).

### 3.4. Secondary Structure Prediction and 3D Structure Polishing and Verification of Multi-Epitope Vaccine Constructs

**Figure 2:**
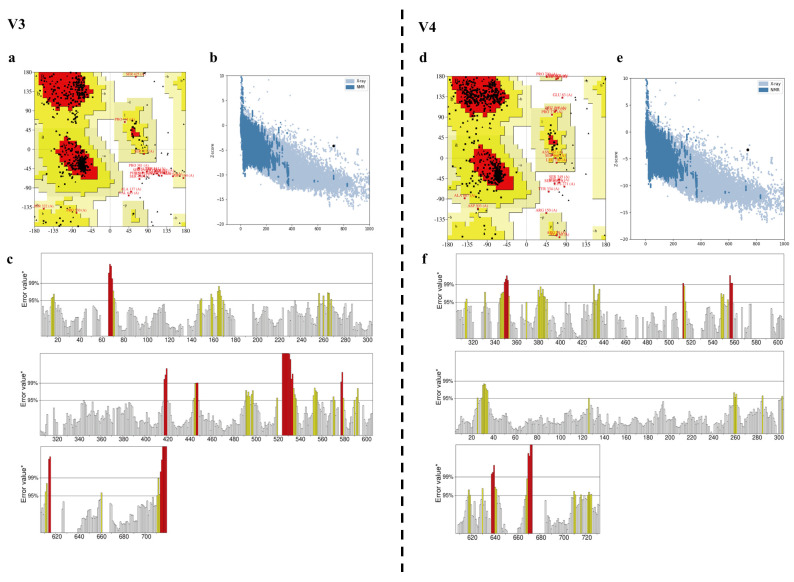
Refinement and validation of the 3D structures of V3 and V4. (**a**,**d**) The Ramachandran plot of the refined 3D structures from the SAVE. Red areas denote the best conformations (V3: 84.4%; V4: 83.5%), yellow areas denote allowed conformations (V3: 12.4%; V4: 13.1%), and white areas denote disallowed conformations (V3: 0.6%; V4: 2.4%). (**b**,**e**) presents the Z-score plot of the refined 3D structures from the ProSA-web server. (**c**,**f**) shows the ERRAT score of the refined 3D structures from the ERRAT server.

The secondary structures of the vaccine constructs were predicted using PSIPRED 4.0 online software, with results indicating that for V1 and V3–V6, alpha helices, beta sheets, and random coils accounted for 20–30%, 10–20%, and 50–60%, respectively (Figure S2). The 3D structures were initially generated via the I-TASSER server using C-scores (range: −5 to 2). The GalaxyRefine server was used to select the optimal refining structure (Figure S3). The 3D structure was validated using the SAVE server based on ERRAT and Ramachandran analyses (Figure 2). V3 and V4 were selected because they had the highest comprehensive scores (Table 3) for antigenicity (>1), optimal solubility (>0.4), and ERRAT score (>85) compared to V1, V5, and V6.

### 3.5. Conformational B-Cell Epitope Prediction

There were four discontinuous B-cell epitopes with 363 residues in V3, which had scores ranging from 0.613 to 0.777 (Figure S4 and Table S3). V4 had four discontinuous B-cell epitopes—also with 363 residues—which had scores ranging from 0.634 to 0.795 (Figure S4 and Table S3).

### 3.6. Analysis of Molecular Docking of Vaccine Constructs with EQCA

#### 3.6.1. Molecular Dynamics Simulation Analysis

**Figure 3:**
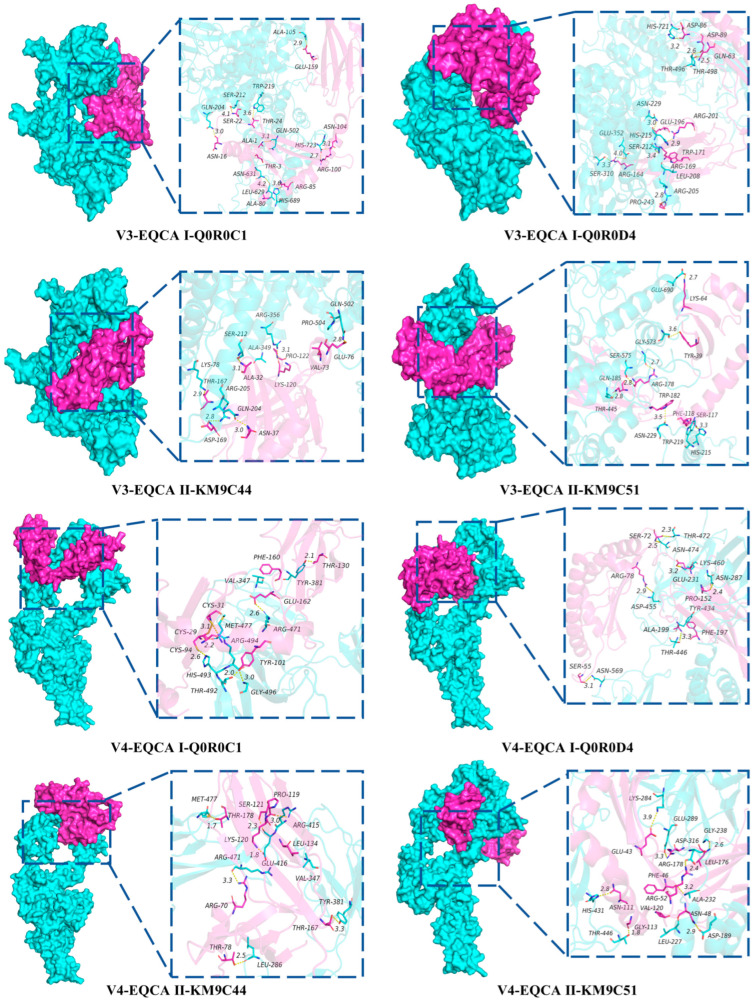
The binding mode of the V3 and V4 complexes with EQCA I/II.Magenta represents the multi-epitope vaccine constructs V3 and V4, and blue represents EQCAI and EQCAII.

Molecular docking analysis revealed substantial binding affinities between EQCA proteins and the V3 and V4 constructs, with computed binding energies of −329.16 kcal/mol and −300.36 kcal/mol (V3/V4-EQCA I-Q0R0C1); −341.17 kcal/mol (V3/V4-EQCA I-Q0R0D4); −285.75 kcal/mol and −292.15 kcal/mol (V3/V4-EQCA II-K9MC44); and −325.24 kcal/mol and −305.03 kcal/mol (V3/V4-EQCA II-KM9C51). Detailed interfacial analysis demonstrated that the contact residues formed multiple stabilizing interactions, including hydrogen bonding networks, electrostatic complementarity (salt bridges), and hydrophobic packing (Table S4). These synergistic intermolecular forces collectively enhanced the structural integrity of the V3/V4-EQCA complexes. Furthermore, surface complementarity assessment revealed steric and electrostatic compatibility between the interacting domains, suggesting favorable geometric alignment for maintaining stable conformational states (Figure 3).

#### 3.6.2. Interaction Between Vaccine Constructs and EQCA

**Figure 4:**
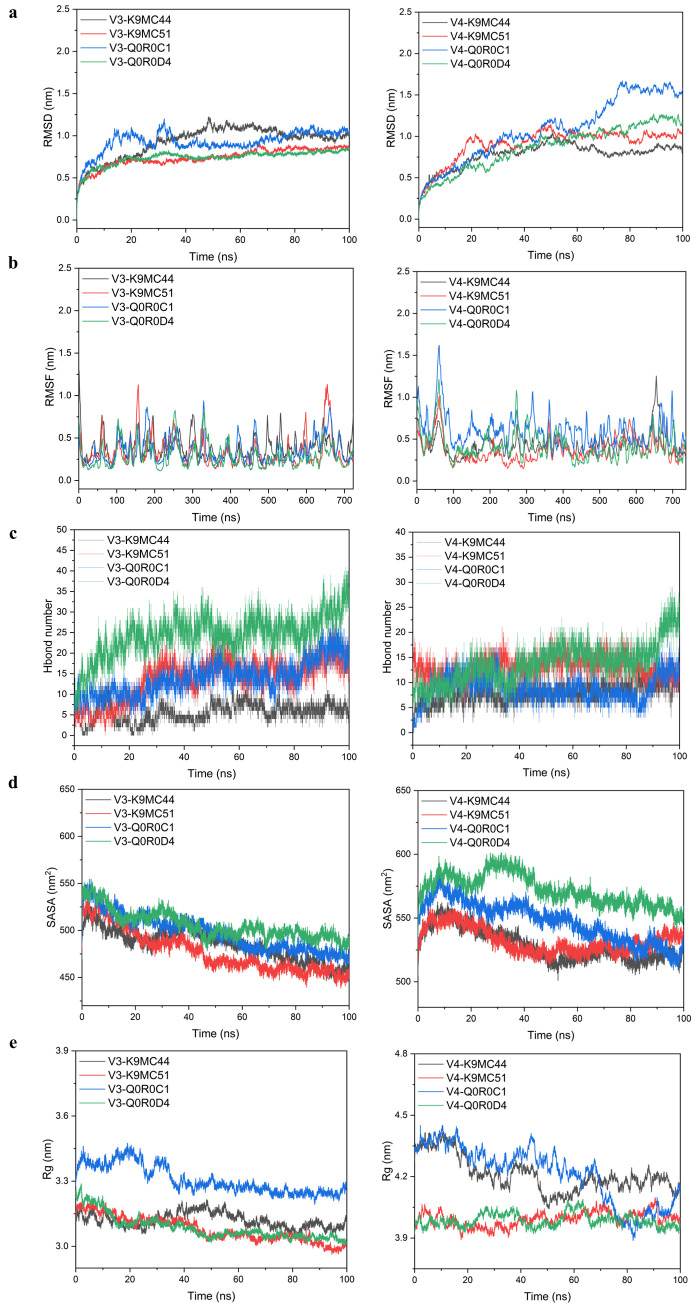
Results of molecular dynamics simulations of V3/V4 with EQCA I/II. (**a**) The RMSD plot created during molecular dynamic simulations for protein complexes. (**b**) The RMSF plot created during molecular dynamic simulations for protein complexes. (**c**) The hydrogen bond number between proteins. (**d**) The SASA plot created during molecular dynamic simulations for protein complexes. (**e**) The Rg changes in protein complexes during molecular dynamics simulations.

The RMSD plot shows that the average RMSD values of the V3-EQCA I-Q0R0D4, V3-EQCA I-Q0R0C1, V3-EQCA II-KM9C44, and V3-EQCA II-KM9C51 proteins were less than 1 nm, while those of the V4-EQCA I-Q0R0D4, V4-EQCA I-Q0R0C1, V4-EQCA II-KM9C44, and V4-EQCA II-KM9C51 proteins were less than 1.5 nm. The complexes reached equilibrium after about 80 ns, which indicates that the proteins were well matched with the proteins and could form stable complexes. According to the RMSF diagram, a few amino acids in the complexes formed by protein–protein interactions underwent significant changes in conformation, mainly because the amino acids in this part of the complex are located in the hinge region of the protein, which is more flexible, and its conformation is prone to a certain degree of change in the simulation process. The Rg plots show that there were significant decreases in the Rg of the V3-EQCA I-Q0R0D4, V3-EQCA I-Q0R0C1, V3-EQCA II-KM9C44, V3-EQCA II-KM9C51, V4-EQCA I-Q0R0C1, and V4-EQCA II-KM9C44 complexes, possibly because after the molecular dynamics simulations, the binding of the proteins to the compounds prompts the proteins to maintain more electrostatic and hydrophobic contacts, which promotes the stability of the complexes. The SASA plots show a significant reduction in the accessible surface areas of the complexes, primarily due to enhanced protein-protein interactions. These reductions in the polar surface area suggest increased protein stability and firmness. Additionally, the hydrogen bonding network diagram reveals that over 10 hydrogen bonds formed between protein amino acids (Figure 4).

#### 3.6.3. Thermodynamic Properties of Vaccine Constructs–EQCA Complexes

Calculating the binding free energy is fundamental for analyzing changes in ligand binding patterns, as it measures the thermodynamic properties of the ligand. Negative binding free energy (ΔG_binding_) values indicate that the system is stable, while positive values indicate instability. Electrostatic interactions and Van der Waals energy (VDWE) effectively stabilize protein–protein interactions. For V3-EQCA I-Q0R0D4 and V4-EQCA I-Q0R0D4 proteins, the lowest binding free energies were −372.72 ± 11.55 kcal/mol and −241 ± 12.68 kcal/mol, respectively. The energy contributions from electrostatic interactions were −239.97 ± 4.6 kcal/mol and −157.18 ± 6.78 kcal/mol, respectively, which were significantly higher than other energy contributions. This suggests that hydrogen bonding plays a crucial role in stabilizing protein interactions. Since the two proteins are more tightly bound, the EVDW interaction contributions are also more pronounced at −241.66 ± 9.79 kcal/mol and −183.22 ± 10.13 kcal/mol, crucial for their stability (Table S5).

### 3.7. Molecular Cloning, Expression, and Purification of the Vaccine Constructs

The lengths of the optimized codon sequences of V3 and V4 were 2177 bp and 2211 bp, respectively, while the average GC content of the adapted sequences was 61.79% and 61.83%, respectively. The codon-optimized sequences were cloned into the pET-30a(+) vector using SnapGene software v.6.2.0, resulting in the construction of the V3–pET-30a(+) and V4–pET-30a(+) recombinant plasmids (Figure S5).

**Figure 5:**
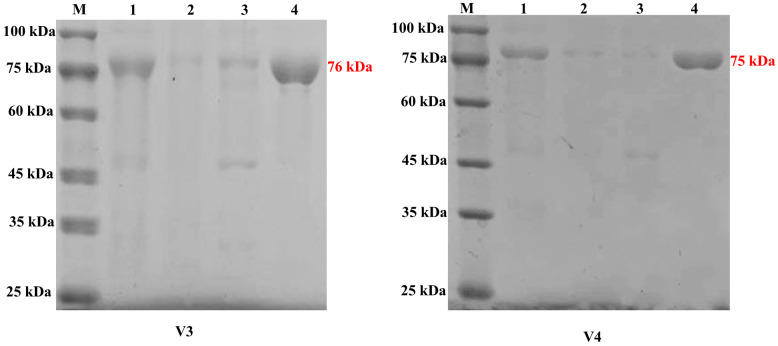
The induced expression of multi-epitope vaccines detected via SDS-PAGE. Due to variations in the blue shade of the photographic images, they were uniformly converted to grayscale for display. M: 180 kDa protein marker; 1: the protein lysate obtained using 8 M of urea buffer; 2: the flow-through fluid from the His-tag Purification Resin; 3: the wash-through fraction from the His-tag Purification Resin using 20 mM of imidazole; 4: the purified protein eluted with 500 mM of imidazole.

Prokaryotic expression of V3 and V4 recombination was carried out using *E. coli* BL21(DE3), and the results of the SDS-PAGE analysis revealed that the V3 and V4 recombinant proteins showed specific bands in the range of 75–100 kDa, which were consistent with the predicted molecular weights of 76 kDa and 75 kDa, respectively (Figure 5 and Figure S6).

### 3.8. Evaluation of the Immunogenicity of Multi-Epitope Vaccines

**Figure 6:**
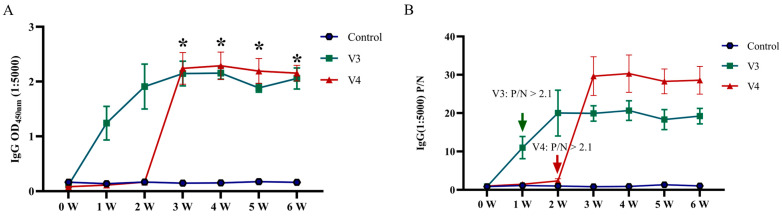
Serum IgG antibody levels pre- and post-immunization with multi-epitope vaccines. (**A**) IgG OD_450nm_ value in serum; (**B**) IgG antibody P/N value in serum (OD_450nm_ in the immune group/OD_450nm_ in the control group) compared with the control group; * *p* < 0.05.

Serum antigen-specific IgG levels at each pre- and post-immunization time point were measured using an ELISA, with all serum samples diluted to a ratio of 1:5000. The results show that serum antigen-specific IgG levels began to rise significantly within 1 week after the prime immunization in the V3 group and within 2 weeks after the prime immunization in the V4 group. Further calculation of the serum antibody positive/negative (P/N) ratio showed that the P/N ratio exceeded the threshold of 2.1 at 1 week post-prime immunization for the V3 group and at 2 weeks post-prime immunization for the V4 group. By week 6 post-prime immunization, the P/N ratios of all immunization groups were above 15.0. These data demonstrate that the V3 and V4 multi-epitope vaccine constructs can induce robust antigen-specific humoral immune responses in BALB/c mice (Figure 6).

## 4. Discussion

Against the backdrop of there being no commercially available vaccines against *R. equi* and the growing spread of drug-resistant strains, existing vaccine research and development strategies have notable limitations: only Vaps family proteins have shown promise as vaccine candidates, but they require further development [40,41]; while inactivated whole-cell vaccines can induce broad-spectrum immunity, they are associated with drawbacks, including severe adverse effects and unstable immunogenicity [8,9,10,11]. Multi-epitope vaccines can precisely integrate core immunogenic epitopes from multiple virulence proteins while avoiding the non-protective antigens of whole-cell vaccines and the immune escape risk of single-antigen formulations, providing a novel direction for *R. equi* vaccine development. Based on five previously validated immunogenic proteins of *R. equi*, we constructed multi-epitope vaccine candidates via bioinformatics-based screening [20,21]; these vaccines present a safer and more efficient design strategy. Consequently, we aimed to design a multi-epitope vaccine against *R. equi*, contributing to the advancement of *R. equi* vaccine development.

Furthermore, the immunogenicity of veterinary vaccines, particularly multi-epitope constructs with limited intrinsic immunogenicity can be significantly enhanced through targeted adjuvant incorporation or structural modification strategies [25]. Previous studies have shown that aqueous-based nanoparticles can be used as adjuvants for *R. equi* vaccines, but such vaccines only induce humoral immune responses and have limited efficacy in clearing intracellularly parasitic *R. equi* [42,43]. In the present study, we selected two bacterial-derived adjuvants, HBHA and L7/L12 ribosomal proteins, to enhance the immunogenicity of our *R. equi* multi-epitope vaccine constructs. These adjuvants were chosen based on their documented ability to activate both innate and adaptive immune pathways in veterinary pathogens. The HBHA protein adjuvant is an immunodominant antigen derived from Mycobacterium species, known to specifically stimulate CD4^+^ T-cells and promote the production of IFN-γ [44]. It is a key pro-inflammatory cytokine critical for controlling intracellular pathogens like *R. equi* L7/L12 ribosomal proteins are conserved bacterial ribosomal components essential for the initiation, elongation, and termination of protein translation, with documented immunogenicity across multiple veterinary pathogens [45]. Critically, these proteins have been shown to activate innate immune receptors (e.g., TLR4) and induce both humoral and cellular immune responses, making them ideal adjuvants for intracellular bacterial vaccines. Through the incorporation of the PADRE (Pan DR Epitope) sequence, a universal CD4^+^ T-cell epitope was designed to further enhance CD4^+^ T-cell activation efficiency by binding to a broad range of MHC class II molecules, including equine EQCA-II [25]. However, it is important to note that this effect remains a theoretical prediction, as CD4^+^ T-cell responses were not experimentally evaluated in this study. Adding rigid linkers with the EAAAK sequence to the C and N termini of adjuvants enhanced structural stability. Rigid connectors facilitated the formation of α-helical structures, which are stiffer and more stable due to the close arrangement of hydrogen bonds and the backbone [46].

An optimized multi-epitope vaccine should incorporate multiple B- and T-cell epitopes to induce robust pathogen-specific immunity [47]. The 27 MHC-I epitopes and 9 MHC-II epitopes screened in this study were highly antigenic, nontoxic, and nonsensitizing. Conservativeness analysis was conducted to ensure their broad-spectrum protective potential against the different strains. A total of six candidate vaccines were designed in this study. V2 was excluded due to a solubility prediction score below the pre-set threshold (<0.4), while V1, V5, and V6 were eliminated due to low ERRAT scores (<85). The screened V3 and V4 vaccines have the potential for stable recombinant expression (molecular weight of 75–76 kDa, instability index of <40, and ProSA Z-score within the reasonable range for natural proteins) and show strong binding affinity to EQCA molecules. Therefore, these two candidate vaccines were selected for subsequent experimental validation. The molecular docking results show that the average binding energies of V3 and V4 to horse-derived immunoreceptors (EQCA I/II) were −335.17/−320.77 kcal/mol and −305.50/−298.60 kcal/mol, respectively. The binding energies were mainly dependent on hydrogen bonding, salt bridging, and hydrophobic interactions. Molecular dynamics (100 ns) verified the solidity of the complex, with RMSD values equilibrating after 70 ns (<1 nm), indicating that V3 and V4 were dynamically stable in conformation upon binding to the receptor. This result is consistent with the kinetic studies of a multi-epitope vaccine against the monkeypox virus, emphasizing the critical role of the hydrogen bonding network in maintaining complex stability [48].

This strategy is similar to the design of a previously developed multi-epitope vaccine against *Mycobacterium tuberculosis*, which significantly enhanced protective efficacy through the synergistic activation of humoral and cellular immunity [49]. In addition, the effectiveness of a vaccine design is determined by its ability to be successfully expressed experimentally; therefore, codon optimization was employed. The codon-optimized vaccine sequences had CAI indices of 0.92 and 0.88, with GC contents of 61.79% and 63.77%. This indicates efficient translation in the *E. coli* expression system, laying the foundation for future recombinant protein preparation.

In this study, we systematically identified and designed multi-epitope vaccine candidates against *R. equi* using bioinformatics approaches, with all screening and design predictions derived from in silico analyses. Although we employed multiple quality control strategies—including cross-validation with various prediction tools and stringent cut-off thresholds—to maximize computational reliability, the inherent limitations of in silico predictions remain, as these analyses cannot fully replicate the complexity and dynamic regulation of the in vivo immune system. Additionally, we only detected antigen-specific IgG responses via an ELISA and did not perform cellular immunity-related validations, including IFN-γ detection and T lymphocyte proliferation assays, nor did we conduct mouse challenge assays to evaluate the in vivo protective efficacy of the vaccines. An additional critical limitation should be noted: the vaccines were designed based on equine MHC molecules (EQCA-I/EQCA-II), but immunogenicity was evaluated in BALB/c mice. There are intrinsic differences in epitope binding preference between murine MHC and equine MHC, and the immune system maturity and immune response patterns of *R. equi*-susceptible foals also differ substantially from those of mice. Therefore, the IgG responses observed in mice cannot be directly equated with immune-protective effects in foals. Subsequent studies should include equine cell-based experiments or in vivo foal trials to verify the immunogenicity and protective efficacy of the candidate vaccines in the target host species.

## 5. Conclusions

In conclusion, we designed two multi-epitope vaccine candidates (V3 and V4) targeting *R. equi* through a systematic bioinformatics pipeline incorporating molecular docking, kinetic simulations, and in silico validation. These candidates showed favorable antigenicity and structural stability in computational analyses, and preliminary experimental validation confirmed they could induce robust antigen-specific IgG responses in BALB/c mice. However, it should be noted that the current findings represent preliminary immunogenicity data only: cellular immune responses and in vivo protective efficacy have not been verified, and the translational gap between murine and equine immunity remains to be addressed. This study provides a valuable reference for the development of multi-epitope vaccines against *R. equi* and lays a theoretical foundation for subsequent experimental validation in equine models.

## Figures and Tables

**Table 1:** Predicted epitopes from 5 vaccine targets of *R. equi*.

Protein	MHC-I Epitopes	MHC-II Epitopes
ABC transporter (WP_013414996.1)	ASDPRVREA, DPSTVVFTL, EAGDVGYVY, GLNEPGAQRRAL, GTHTQVWKVTV, QVLNQREGR	AAIYDVLMRYDTATKEYQ, EAIVKALNPETFNERG
PBD2 (WP_013414386.1)	ANAPVTGFY, VAYDELTAK, GTSTTLENYNGTTC, VSPEVASTL, GSDNRLFSK	VPTDDRYKYLRVYPPNPAAP, TIANGGMRMESHLVS
NlpC/P60 (WP_013416802.1)	AYREAGVEL, SGMGFHGFY, VPYQWGGTT, VAADAAVTK	VSISAIRTSNSGMGFHGF
Esterase (WP_013415053.1)	ATNECTHRL, KADDALHGV, LTQELPPVIDSAL, TSKAAPTLYLL	LKGGPKLYVANASGLPG, QGINGNVSTLANQIIVGG, VFNLALKDPQLYKAVG
M23 (WP_013415127.1)	GPHLHFEVWSPGGA, SADAVLIEL, VLIELDPTL, QSTGPHLHF, EIPAELPQV, AGPASGFGL, YQVNVGQHV	SADAVLIELDPTLRRGRHRE

**Table 2:** The antigenicity, immunogenicity, allergenicity, toxicity, solubility, and physicochemical properties of the constructed vaccines.

Vaccine Construct	Antigenicity	Allergenicity Toxins	Immunogenicity	Solubility	Half-Life (hours)	Instability Index	Aliphatic Index	GRAVY
V1	1.15	none	7.85	0.45	>10	33.65	70.44	−0.31
V2	1.25	none	3.24	0.39	>10	31.86	65.06	−0.29
V3	1.14	none	4.78	0.54	>10	28.96	71.89	−0.18
V4	1.18	none	6.75	0.44	>10	35.99	71.03	−0.29
V5	1.30	none	2.14	0.41	>10	34.60	65.63	−0.26
V6	1.17	none	3.68	0.55	>10	31.30	72.55	−0.15

**Note:** Proteins with antigenicity greater than 0.5 and instability coefficient of less than 40 are considered highly antigenically stable, and those with GRAVY mean of less than 0 are considered hydrophilic.

**Table 3:** C-scores, Z-scores, and ERRAT scores of vaccine constructs.

	V1	V3	V4	V5	V6
C-score	−2.23	−0.74	−2.28	−1.22	−1.16
Z-score	−5.06	−4.18	−3.34	−4.48	−3.43
ERRAT	63.36	87.82	88.62	55.58	68.40

**Note:** C-scores in the range [−5, 2] reflect model quality confidence, with higher values indicating greater confidence. ERRAT score assesses model quality via atom interactions, and high ERRAT scores denote good quality. Low Z-scores indicate a high-quality model with structural similarity to natural proteins.

## Data Availability

The original contributions presented in this study are included in the article/Supplementary Material. Further inquiries can be directed to the corresponding author(s).

## Supplementary Materials

The following supporting information can be downloaded at: https://www.mdpi.com/article/10.3390/vetsci13070655/s1, Figure S1: Flow diagram of the multi-epitope vaccine design in this study; Figure S2: Secondary structure of multi-epitope vaccine constructs; Figure S3: 3D structures of the vaccine constructs; Figure S4: ElliPro tool for V3 and V4 conformational B cell epitope analysis results; Figure S5: The multi-epitope vaccine was computer cloned into the pET-30a(+) expression vector; Figure S6: Raw Uncropped Image of SDS-PAGE of Induced Expression of the Multi-Epitope Vaccine. Due to variations in the blue shade of the photographic images, they were uniformly converted to grayscale for display. M: 180 kDa protein marker, 1: the protein lysate obtained using 8 M urea buffer, 2: the flow-through fluid from the His-tag Purification Resin, 3: the wash-through fraction from the His-tag Purification Resin using 20 mM imidazole, 4: the purified protein eluted with 500 mM imidazole. (a) SDS-PAGE results for V3. (b) SDS-PAGE results for V4. As SDS-PAGE of V4 was performed simultaneously with other protein samples, all results are presented on the same gel. The band labeled V4 in the figure corresponds to the electrophoresis result of the V4 protein; Table S1: Antigenic epitopes; Table S2: Assembly of multi-epitopes vaccine constructs; Table S3: Predicting conformational B-cell epitopes of V3 and V4; Table S4: The docking results of two target protein; Table S5: Protein–protein docking score of V3 and V4 by HDOCK.
