# Supplementary material for: Designing a Multi-Epitope Vaccine Candidate Against Rhodococcus equi Based on the Bioinformatics Technique
Source: Vet Sci. 2026 Jul 7;13(7):655. doi: 10.3390/vetsci13070655 (PMC13417819; doi:10.3390/vetsci13070655)
Supplement: Supplementary file 1 [file vetsci-13-00655-s001.zip › Fig S.2 Secondary structure of multi-epitopes vaccine constructs.pdf]

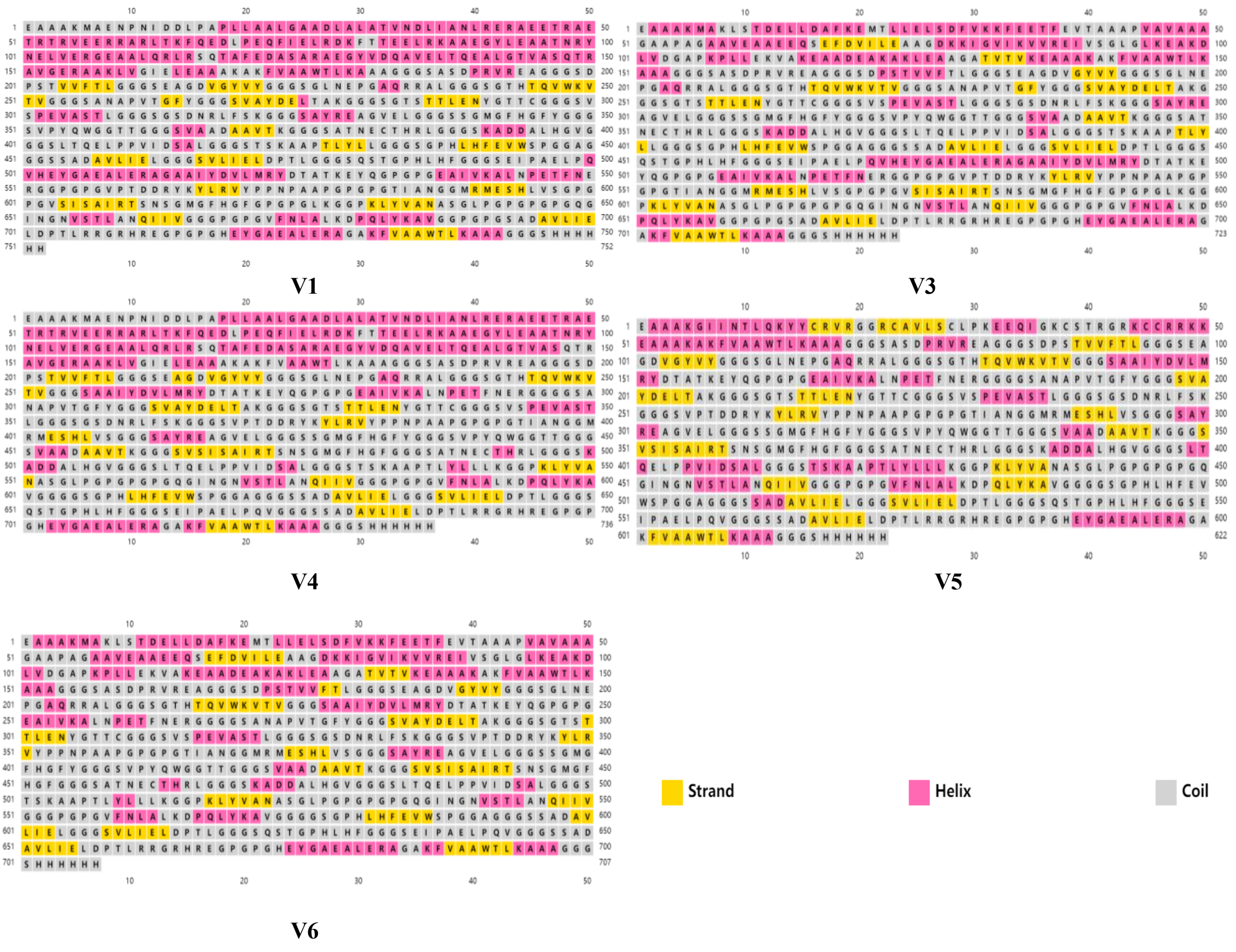
V1
V3
V4
V5
V6
Strand
Helix
Coil
