# Supplementary figures and images for: Designing a Multi-Epitope Vaccine Candidate Against Rhodococcus equi Based on the Bioinformatics Technique
Source: Vet Sci. 2026 Jul 7;13(7):655. doi: 10.3390/vetsci13070655 (PMC13417819; doi:10.3390/vetsci13070655)

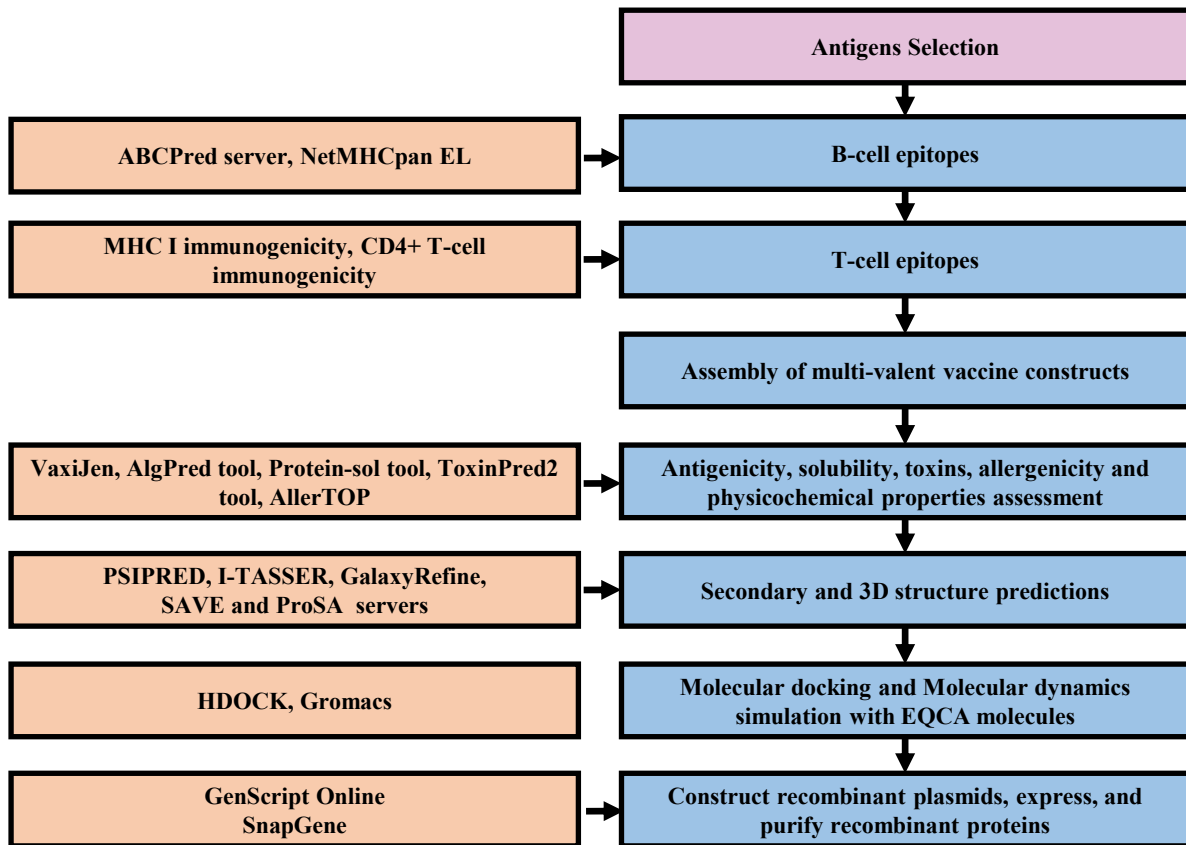

Supplement: Supplementary file 1 [file vetsci-13-00655-s001.zip › Fig S.1 Flow diagram of the multi-epitope vaccine design in this study..pdf]

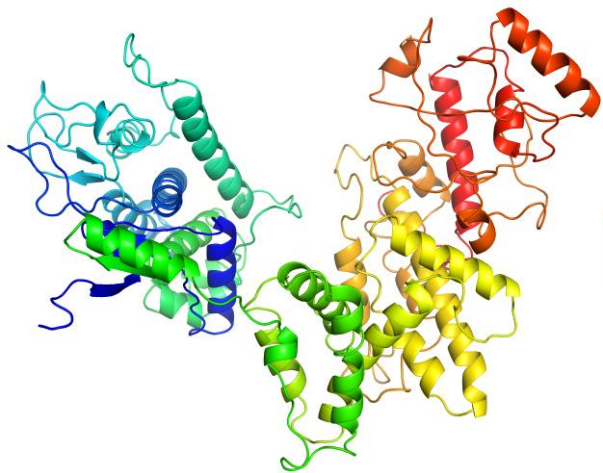

**V3**

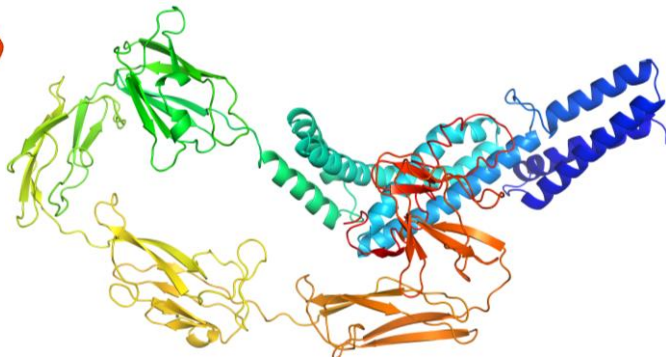

**V4**

Supplement: Supplementary file 1 [file vetsci-13-00655-s001.zip › Fig S.3 3D structures of the vaccine constructs.pdf]

V3

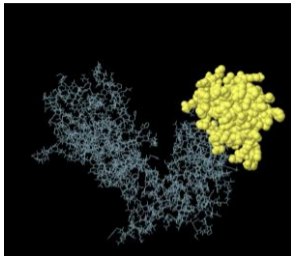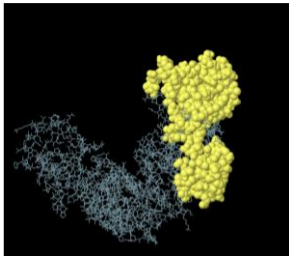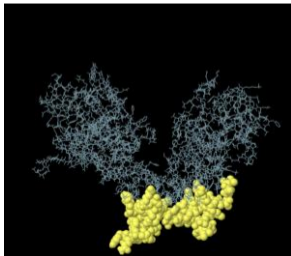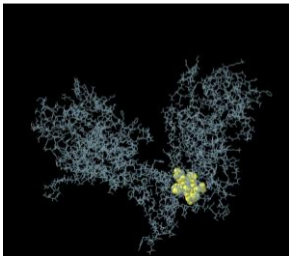

V4

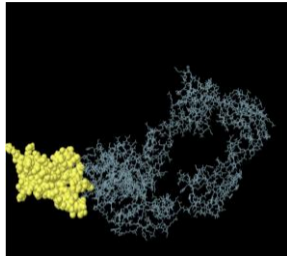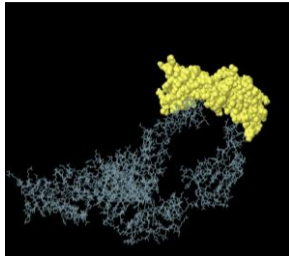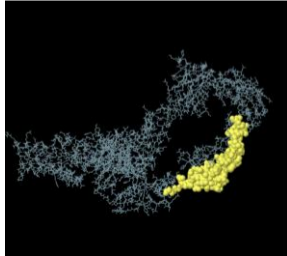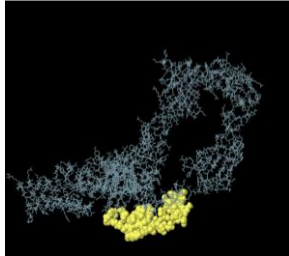

Supplement: Supplementary file 1 [file vetsci-13-00655-s001.zip › Fig S.4 ElliPro tool for V3 and V4 conformational B cell epitope analysis results. Yellow spheres represent conformational B-cell epitopes.pdf]

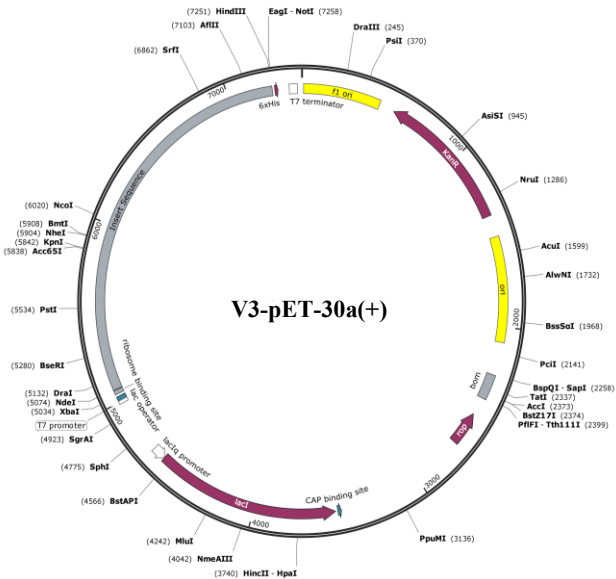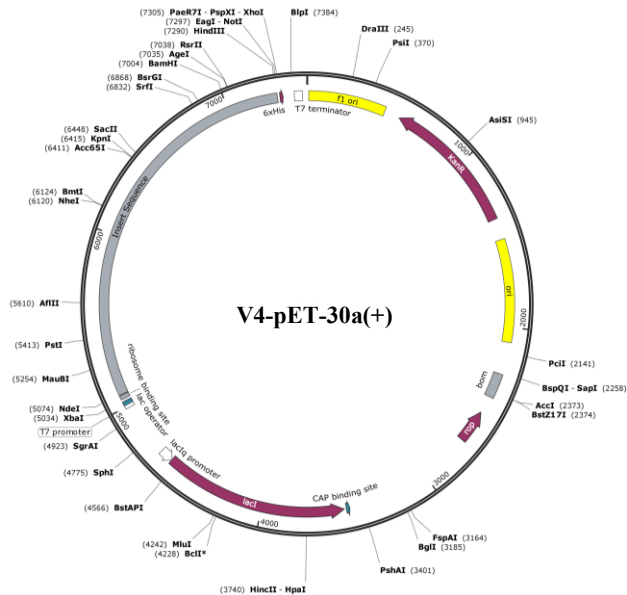

Supplement: Supplementary file 1 [file vetsci-13-00655-s001.zip › Fig S.5 The multi-epitope vaccine was computer cloned into the pET-30a(+) expression vector. The codons of the vaccine are indicated in gray.pdf]

**a**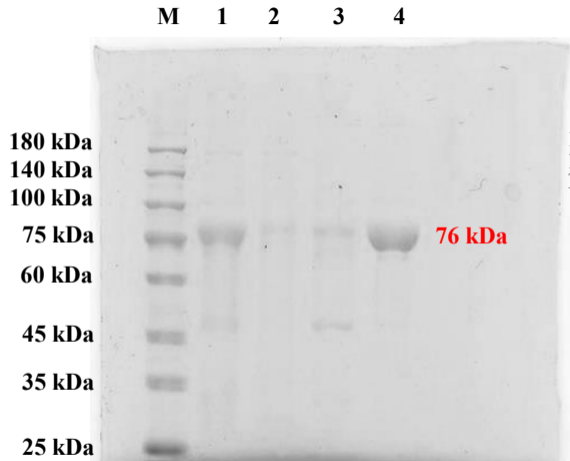**b**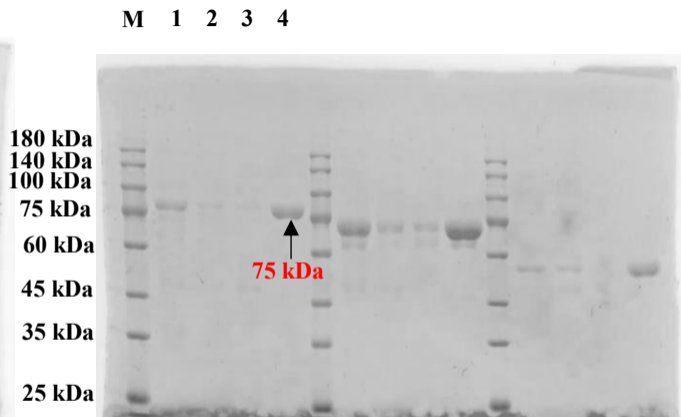

Supplement: Supplementary file 1 [file vetsci-13-00655-s001.zip › Fig. S.6 Raw Uncropped Image of SDS-PAGE of Induced Expression of the Multi-Epitope Vaccine.pdf]
